# Advances in integrative structural biology: Towards understanding protein complexes in their cellular context

**DOI:** 10.1016/j.csbj.2020.11.052

**Published:** 2020-12-03

**Authors:** Samantha J. Ziegler, Sam J.B. Mallinson, Peter C. St. John, Yannick J. Bomble

**Affiliations:** Biosciences Center, National Renewable Energy Laboratory, 15013 Denver West Parkway, Golden, CO 80401, USA

**Keywords:** ISB, Integrative structural biology, NMR, nuclear magnetic resonance, cryo-EM, cryo-electron microscopy, XL-MS, cross-linking mass spectrometry, SAXS, small angle X-ray scattering, SANS, small angle neutron scattering, FRET, Forster resonance energy transfer, EPR, electron paramagnetic resonance, PDB, Protein Data Bank, MX, macromolecular crystallography, MR, molecular replacement, SAD, single-wavelength anomalous dispersion, cryo-EM SPA, cryo-EM single particle analysis, ML, machine learning, MSAs, multiple sequence alignments, cryo-ET, cryo-electron tomography, CLEM, correlated light and electron microscopy, Integrative structural biology, Quinary interactions, Protein structure prediction, Cryo-electron tomography, Cryo-electron microscopy, X-ray crystallography, Crosslinking mass spectrometry, Protein docking

## Abstract

Microorganisms rely on protein interactions to transmit signals, react to stimuli, and grow. One of the best ways to understand these protein interactions is through structural characterization. However, in the past, structural knowledge was limited to stable, high-affinity complexes that could be crystallized. Recent developments in structural biology have revolutionized how protein interactions are characterized. The combination of multiple techniques, known as integrative structural biology, has provided insight into how large protein complexes interact in their native environment. In this mini-review, we describe the past, present, and potential future of integrative structural biology as a tool for characterizing protein interactions in their cellular context.

## 1 Introduction

One of the main goals of molecular biology is to understand cellular processes at the molecular level. Most of these processes are the result of low-affinity interactions between cellular components, e.g. transient protein interactions, termed quinary interactions [1], [2]. Most commonly, to characterize these quinary interactions at the molecular level, the complexes must be purified to homogeneity [3]. On one hand, this purification process removes the proteins from the noise of their native environment, and the stringent steps of purification tend to eliminate weak protein interactions [4]. On the other hand, studying proteins *in situ,* using techniques such as confocal microscopy and super-resolution microscopy, can lead to a loss of resolution [5], [6]. Thus, only subcellular protein localization and protein-protein co-localization can be determined instead of atomic structures. Therefore, molecular biologists have started combining multiple techniques to contextualize low-resolution protein co-localization with high-resolution structural information resulting in a field commonly referred to as integrative structural biology (ISB) [7], [8], [9].

**Fig. 1:**
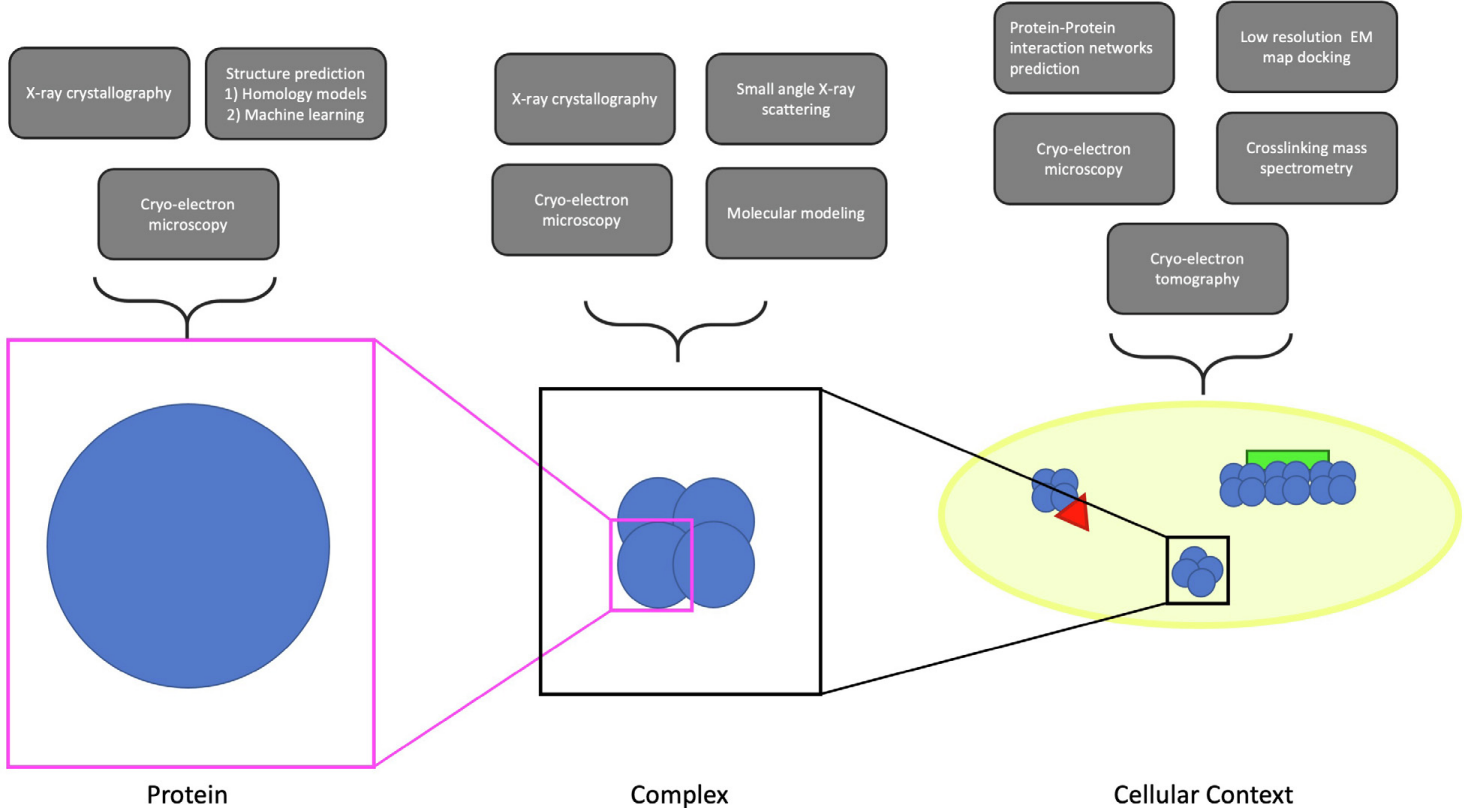
Determining protein interactions in their native environment. The determination of the structure of a protein (blue) complex in its cellular milieu necessitates the combination of many techniques at different scales. Capturing complexes in their native environment is essential to fully understand their role in the cell (yellow) and the interactions that exist with other cell components (*e.g* another protein (red) or a scaffold (green)) leading to alternate ultra-structures and functions. (For interpretation of the references to colour in this figure legend, the reader is referred to the web version of this article.)

ISB is a catch-all term that generally indicates the combination of a classical structural biology technique and any other technique that gives structural information to form a more complete, higher resolution picture than can be created with classical structural biology alone [7], [8]. Here, classical structural biology refers to either X-ray crystallography, nuclear magnetic resonance (NMR), or cryo-electron microscopy (cryo-EM). Any other technique that gives extra information could be integrated with any of the above structural techniques, such as cross-linking mass spectrometry (XL-MS), small angle X-ray scattering (SAXS), small angle neutron scattering (SANS), molecular docking, machine learning generated structural models, protein mutagenesis, or Forster resonance energy transfer (FRET), among many others. Because these techniques have a wide variety of efficiency and resolution, specific combinations of experiments may work better at different structural scales (Fig. 1). In this review, our goal is to describe first historically used structural biology techniques, followed by novel approaches to ISB that we believe will substantially affect the field in the near future. First, we will briefly describe some well-characterized techniques for structural determination of isolated proteins and complexes. We will then discuss the progression of ISB over the past twenty years to characterize stable or transient protein complexes *in vitro*. Finally, we will discuss how a combination of techniques and recent advances in cryogenic electron microscopy can enable the characterization of quinary interactions *in situ*. Table 1 provides a summary of the more common techniques that can be combined successfully in ISB.

**Table 1:** Summary of techniques described in this review and their contributions in ISB.

	Technique	Description in the context of ISB	References
*Structural characterization of proteins*			
Common technique	Macromolecular crystallography	• Captures atomic resolution detail of stable protein conformations • Data can be fit into SAXS/molecular docking/low-resolution cryo-EM data	[15]
	NMR	• Captures atomic detail of small, flexible proteins • Data can be fit into SAXS/molecular docking/low-resolution cryo-EM data	[22], [24], [25]
	SAXS/SANS	Provides overall protein complex shape that can be fit with atomic structures	[40], [42], [43], [44]
Recent advancement	Cryo-EM SPA	• Captures high-resolution stable and lower-resolution flexible protein conformations that can be fit into SAXS/molecular docking • Data can provide overall shape to be fit with atomic structures	[45], [52]
	Computational modeling	Detailed atomic subunit predictions which can be fit into SAXS/molecular docking or used in MX analysis	[61], [66], [68]

*Identification and characterization of protein*–*protein interactions*			
Common technique	Co-IP	Isolates strong protein interactions using affinity pulldowns that can be characterized by MS	[84]
	FRET	Determines domain positioning or how two proteins interact based on proximity of two fluorophores	[101]
Recent advancement	XL-MS	Captures strong and weak interacting partners and identifies which residues are in proximity to each other	[85], [115], [116], [117]
	Molecular docking	Uses structural data and surface predictions to determine how protein complexes interact	[87], [88], [89], [90]
	Proximity labeling	Identifies proteins that come within 10 nm of the protein of interest	[86]

*Contextualization of protein*–*protein interactions*			
Future of ISB	Whole-cell cryo-ET	• Determines nanometer-scale resolution of how proteins are spatially separated • Provides a snapshot of transient interactions at the time of freezing	[103], [104]
	Single-cell cryo-EM	• Identifies transient and stable protein complexes in a cell • Atomic resolution can be reached if there are multiple copies of the complex	[111], [112]
	XL-MS and cryo-EM SPA	Traps transient and stable protein complexes with crosslinkers which can be characterized with cryo-EM SPA	[102], [114]

## 2 Most common techniques for protein structure determination and new approaches

One of the most common ways to define protein interactions is to first determine the structures of individual components of the complex. Using these pieces, interactions can then be modeled and built into larger structures. In this mini-review, we refer to this approach as bottom-up ISB. In this section, we describe five common approaches to individual protein structure determination, starting with more traditional methods such as X-ray crystallography, SAXS, and NMR and finishing with methods that have become feasible in the past decade such as cryo-EM and computational modeling.

### 2.1 X-ray crystallography

Since the first protein structure – of sperm whale myoglobin – was solved by John Kendrew in the late 1950s using X-ray crystallography [10], the technique has been the preferred method for structural biologists. Of the more than 160,000 structures that have been deposited in the Protein Data Bank (PDB) to date, almost 90% have been solved using X-ray crystallography (https://www.rcsb.org/stats/summary). Advances in every aspect of the workflow required for X-ray crystallography have had a transformative effect on the time required for and complexity of solving a crystal structure. The limiting step of biological macromolecular crystallography (MX) is crystallization. While robotics and a wealth of know-how have drastically sped up the process, it is still considered a ‘dark art’ – the ability to predict crystallization conditions remains elusive, with the crystallization step itself being the most time and labor intensive in solving a structure. Failure in this single step of macromolecular crystallography is often the main reason to look for other techniques to acquire reliable protein structures.

**Fig. 2:**
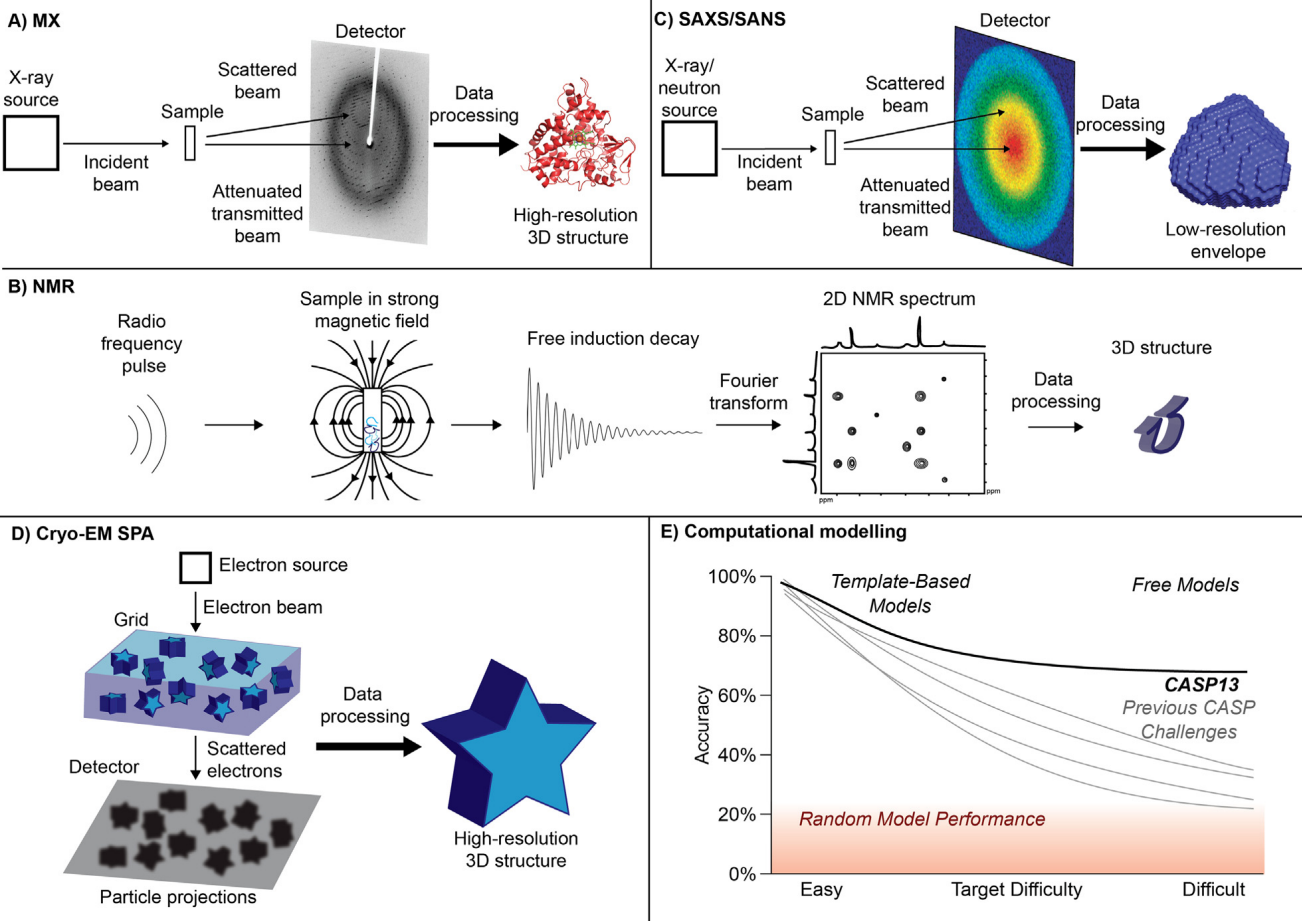
Techniques used for structure determination. A) Schematic representing the MX data collection workflow, with GcoA (5NCB) as the resulting structure. B) Schematic representing NMR data collection workflow, with a 2D NMR spectrum as an example C) Schematic representing the SAXS/SANS data collection workflow. D) Schematic representing the cryo-EM SPA data collection workflow, using a 3-dimensional star as an example sample. E) Trends in recent structure prediction challenges. Previous challenges saw prediction accuracy degrade close to the performance of baseline random models for protein structures without suitable templates. In CASP13, with the rise of ML-enabled free modeling techniques, prediction accuracies tended to remain above 60% even for the hardest of protein targets.

Advances over the last couple of decades have resulted in a current state of technology once a protein has been crystallized and demonstrated to diffract X-rays, a dataset can be collected in seconds due to high brilliance synchrotron beamlines [11]; high resolution, fast readout detectors [12]; and automated and precise sample mounting and manipulation [13]. Once a dataset has been collected, it must be indexed and integrated to determine the unit cell of the crystal – i.e. the dimensions of the smallest repeating unit of which the crystal is comprised. With the crystal lattice information in hand, the phases must be solved. Two methods dominate solving the phase problem in crystallography: molecular replacement (MR) and single-wavelength anomalous dispersion (SAD) (with 70% and 7% of structures solved, respectively) (https://www.rcsb.org/stats). MR uses known homologous structures to compute predicted phases of the unknown structure by placing them in the same position and orientation in the unit cell of the crystal. For this reason, as the number of available structures grows, so does the use of MR to solve the phase problem. SAD is a form of experimental phasing, that is, the phases of the diffraction pattern are measured, rather than predicted. It relies on a breakdown of Friedel’s law (centrosymmetric diffraction spots in a diffraction pattern have equivalent intensities but inverted phase) when the energy of a diffracting X-ray is at the absorption edge of a heavy atom (e.g. 0.9795 Å for Se) within the crystal. For an in-depth review of phasing methods see [14]. This breakdown provides a starting point for determining the phases of the whole structure. Determination of the phases results in an electron density map, from which a model can be built that is proceeded by iterative cycles of phase refinement and further model building (Fig. 2A). Once refinement is complete, validation ensures that the structure does not violate known constraints of biological macromolecules. Nowadays, all of these steps can be heavily automated to the point that amenable structures can be solved with no human intervention whatsoever [15], [16], [17]. The relative trivialization of so many steps in MX has led researchers to develop specialized beamlines to address new bottlenecks in the process. For example at the Diamond Light Source in the UK, VMXi is a fully *in situ* beamline in which diffraction experiments take place in crystallization plates mounted directly onto the beamline [18], and I23 is a long wavelength beamline for *in vacuo* (to reduce background diffraction from air) SAD experiments using native sulfur and phosphorous atoms and has been touted in particular for solving membrane protein structures [19]. With the advent of X-ray free electron lasers (XFEL), time-resolved crystallography is now possible. The much brighter and shorter X-ray pulses provided by XFELs have, for example, allowed the capture of an oxygen intermediate in the catalytic cycle of cytochrome *c* oxidase [20], and have also been shown to ‘outrun’ radiation damage since the diffraction pattern is collected before it can be influenced by the damage the X-rays have caused to the sample [21]. X-ray crystallography’s record for producing structures of single biological macromolecules remains unparalleled. Its major limitation, however, is that these structures are simply snapshots of the protein in a crystalline state which represents a drawback for ISB, wherein the dynamic nature of a biological system must be captured in which case X-ray crystallography can be augmented using other techniques.

### 2.2 Nuclear magnetic resonance spectroscopy

NMR is the second most commonly used technique for determining protein structures after MX, with 7.7% of all structures deposited in the PDB (https://www.rcsb.org/stats). Atomic nuclei with an odd mass number have a quantum mechanical property known as spin that can interact with external magnetic fields and is the basis for NMR [22]. This interaction can be measured by applying a radio frequency pulse to the sample perpendicular to the magnetic field causing nuclear magnetization, which varies depending on the chemical environment of the nucleus as dictated by other atoms in the vicinity. This nuclear magnetization decays over time in what is called free induction decay (FID), and this can be measured as perturbations to the magnetic field. The FID is converted from a function of time to frequency via Fourier transform and is termed chemical shift. Because all active nuclei in a molecule will have slightly different chemical environments, the FID will have a frequency component for each nucleus, and the Fourier transform will in turn produce a unique chemical shift value for each one (Fig. 2B) [23]. Due to the complexity of a protein molecule, an NMR spectrum will have many overlapping peaks. In order to deconvolute these peaks, multi-dimensional NMR is used in which separate pulses are applied to the sample (the number of pulses corresponding to the number of dimensions) with the time between the pulses systematically increased [24]. This can be combined with heteronuclear methods that use proteins labelled with ^13^C or ^15^N isotopes as one or more of the extra dimensions in addition to ^1^H [25]. For example, ^1^H-^15^N heteronuclear single quantum coherence (HSQC) is typically the first NMR experiment performed on a protein and is used to assign peaks to all N atoms with a bonded hydrogen and used for fingerprinting and evaluating whether the expected number of peaks are present, which can indicate the homogeneity and monodispersity of the sample [26]. The chemical shift peak or peaks (certain techniques split chemical shift peaks depending on how the nucleus is bonded to other NMR active nuclei) of each nucleus can give information about various geometric relationships between nuclei by applying different pulse sequences to the sample. These include for example the distances between nuclei via Nuclear Overhauser Effect SpectroscopY (NOESY) [27], the angle of bonds in COrrelated SpectroscopY (COSY) via the Karplus function [28], and relative orientation through Residual Dipolar Coupling (RDC) [29]. Each of these methods produce restraints that can be combined to compute the structure of the target protein. In addition to solving the 3D structure of a protein in solution, NMR is well suited to studying protein dynamics. Since protein dynamics cause changes in the local environment of nuclei, the relaxation time after application of the radio pulse is also influenced, resulting in a change in chemical shift peaks. ^15^N labelling has long been used to study backbone dynamics, and has been supplemented by development of pulse sequences for ^1^H and ^13^C for studying the movements of amino acid side chains. Such dynamics can be studied on the timescale of picoseconds to milliseconds depending on the methods used [30] and an extension of these technologies has also allowed real-time observations of protein folding [31]. Hydrogen-deuterium exchange can also be used to measure the lability of hydrogens bonded to nitrogen, oxygen, or sulfur atoms, by replacing water with heavy water. Since these will readily exchange in water, and deuterium is not an NMR-active nucleus, the change in intensity of a chemical shift peak over time can give indications as to how dynamic a region of a protein is – the less dynamic, the less exposure to the solvent and the slower the rate of H/D exchange [32]. There are major limitations of protein NMR that have made it less popular than crystallography. For example, data collection can take weeks (compared to minutes on synchrotron beamlines) meaning low instrument availability, and that samples must be stable on this timeframe [33]. Another is the upper size limit of the target protein - with conventional methods, this is around 25 kDa. However, deuteration of the sample to reduce the complexity of the 1H spectra, the development of Transverse Relaxation Optimized SpectroscopY (TROSY) pulse techniques, paramagnetic relaxation enhancement (PRE), RDCs and pseudo contact shifts (PCS) has raised this limit towards 100 kDa, though this requires much longer experiment times [34]. While relative and actual numbers of structures deposited to the PDB continue to decline, NMR is finding new applications to maintain its relevance to ISB. For example, much focus has been given recently to NMR of membrane proteins [35], [36], and also to whole- and in-cell NMR of macromolecules [37], [38], [39].

### 2.3 Small angle X-ray and neutron scattering

Small angle scattering (SAS) measures the scattering of radiation by biological molecules. Unlike MX, it derives low resolution (about 10 Å) structural information from disordered molecules in an aqueous environment. This means that SAS can be employed to interrogate the innate dynamic nature of biological macromolecules that is not captured by MX (and for SAXS without lengthy sample preparation). In the case of SAXS, this is achieved by measuring X-rays scattered at low angles (0.1–10°). Because the signal from the scattered X-rays is so weak and the angle so small, SAXS requires high brilliance, collimated radiation, and long sample-to-detector distances. Hence, beamlines are almost always situated at synchrotrons. Since the scattering pattern obtained is from a molecule that is constantly tumbling in solution, it is continuous and radially symmetric, and gives information on the radius of gyration (R_g_), maximum dimension (D_max_), and molecular weight. A model can be built using dummy atoms confined by this experimentally determined R_g_ value, and this model is refined until the calculated scattering pattern of the model matches the observed diffraction pattern, giving an ‘envelope’ of the molecule in solution (Fig. 2C). As this model is of a molecule in solution approximating its natural state, features can be captured that are not possible in MX, where the sample is in a ‘non-natural’ crystalline environment: oligomeric state, protein-protein hetero-interactions, intra-protein domain movements, and disordered proteins or regions of proteins. For an in-depth review of SAS including the determination of these features, see [40]. High resolution structures determined through MX (or others) can be built into these low resolution ‘envelopes’ as a basic form of ISB of individual proteins or protein complexes. Aside from the low resolution, SAXS is not without downsides – for example, dilute samples (meaning the concentration is low enough that essentially no non-specific inter-particle interactions take place) and monodisperse samples (meaning all the particles are identical at the relevant resolution) of high purity are required to ensure necessary data quality. The mild experimental conditions that lend biological relevance also leave the sample exposed to radiation induced damage – unlike MX which is typically performed at cryo temperatures to mitigate this problem. Radiation protection can be supplied to the experiment in the form of free-radical scavenging species (such as DTT or TCEP), and for extreme cases where this is insufficient, cryo-SAXS has been developed [41]. Additionally, even if high quality data is collected, multiple theoretical 3D models can be fit to a given 2D scattering pattern, meaning without additional experimental data to back up a model, conclusions from SAXS data alone must be drawn with caution.

Small angle neutron scattering (SANS) operates in a similar manner to SAXS, with the main difference arising from the way in which the radiation used to analyze the sample interacts with nuclei. While X-ray scattering intensity by electrons is in correlation with the number of electrons (and by extension the atomic number of the elements within the sample), the interaction of neutrons with atomic nuclei is dependent on the nucleus type and is not correlated with atomic number of the element. This has two consequences relevant to biological SANS. First, common biological elements such as carbon, nitrogen, and oxygen are similarly visible to neutron scattering as heavy atoms. Second, and more importantly, scattering (specifically the scattering length density, or SLD) varies depending on the isotope present. For isotopes of the above-mentioned biological elements the difference is too small to exploit, but the SLDs for hydrogen (H) and deuterium (D) are drastically different [42]. This allows, for example, the highlighting of a single molecule within a dimer by deuterating one of the partners and leaving the other protonated. Since the observed scattering intensity of a sample is related to the difference between the SLD of the sample and that of the solvent, matching the SLD of the solvent (by mixing H_2_O and D_2_O in varying ratios) and a specific component of the sample (termed contrast matching) allows the removal of that component from the scattering pattern [43], [44]. In turn, this permits the individual contributions of each component of a system to be analyzed. SANS comes with limitations that correspond to its advantages. Deuteration of samples can be expensive and time consuming (though potentially less so, and more predictably, than crystallization for MX). Neutron scattering is also weaker than that of X-rays, meaning that a greater sample concentration must be used than for SAXS, and care must be taken to ensure that this does not give rise to non-specific interactions. Additionally, neutron sources are less widely available than X-ray sources and access to instrumentation can be more difficult, meaning SANS experiments will normally only be pursued when SAXS is unable to answer the questions posed by a research project.

### 2.4 Single-particle Electron Microscopy

The recent resolution revolution in the field of cryo-EM single particle analysis (cryo-EM SPA) has provided an alternative to X-ray crystallography for large (>100 kDa), flexible molecules [45]. Although cryo-EM has been used since the 1980s, the structures that were generated were largely limited to lower resolution (>15 Å), leading to the field being scorned as “blobology” [46]. However, in the early 2010s, several significant advances in both technology and data processing algorithms led to the high-resolution structure of the transmembrane channel TrpV 1 in 2013 [47]. These advances can be separated into three categories: 1) the development of the direct electron detector, which directly records movies of the positions of the electrons, allowing for correction of beam induced particle motion [48], 2) the increase in computational power available for data analysis and the development of algorithms for data processing [49], and 3) the innovation of the Volta phase plate, which allows for in-focus imaging of cryo-EM samples [50]. Ever since the publication of the TrpV 1 structure, there has been an exponential increase in cryo-EM structures deposited in the PDB, especially for proteins that had previously proved recalcitrant to traditional X-ray crystallography methods (https://www.emdataresource.org/statistics.html). Cryo-EM has been especially promising for membrane proteins, due to the small amounts of sample needed for a high-resolution structure. Each year, the high-resolution limit of cryo-EM has been pushed closer towards atomic detail, as the equipment and data processing algorithms continue to improve. Currently, the highest resolution structure is the 1.22 Å reconstruction of mouse apoferritin [51]. One of the most intriguing aspects of cryo-EM is that proteins are captured in a near-native environment in a layer of vitreous ice [52]. Without forcing proteins into a stable conformation to form a crystal lattice, multiple conformations of the protein can be captured in a single collection of data (Fig. 2D). These conformations can be separated and analyzed *in silico* by a variety of programs. For example, cryoDRGN is a neural network used to model reconstructions along a continuous spectrum of protein movement [53] and 3D Variability Analysis in cryoSPARC can be used to analyze both discrete and continuous protein conformations [54]. Further, samples can be frozen in milliseconds, which allows for time-resolved cryo-EM that captures enzyme transitions and intermediate states, as evidenced by structures that capture the movements of the ribosome, for example [55], [56], [57].

While the field of cryo-EM SPA has grown exponentially, it is not a panacea for all structural biology problems. Currently, the rate-limiting step for most SPA cryo-EM is grid preparation. Samples that are too heterogeneous require extra purification or optimization of sample conditions to be analyzed at high-resolution. The blotting in plunge freezing is also a problem due to its high variability, even when working on the same day – resulting in variable ice thickness that limits sample acquisition to only portions of a grid [58]. Blotting also inherently wastes samples, wicking away over 99% of the drop applied to the grid. Finally, particles tend to settle at the air–water interface, which can result in protein denaturation, depending on the stability of the sample [58]. Multiple companies have begun to address the problems inherent in plunge freezing and blotting by designing blot-free freezing methods, such as Chameleon (developed from Spot-It-On) [59] and the Vitrojet [60]. The Chameleon utilizes self-wicking grids combined with samples that are applied via piezo electric dispenser to create a reproducible and uniform sample thickness on the grids without blotting. The grid is then plunge frozen in the same manner as the established procedure [59]. In contrast, the Vitrojet has been developed to avoid using specialized grids, focusing on using capillary action between a pin coated in sample and the grid, which deposits a thin strip of protein on the grid. The grid is then jet vitrified with a blast of liquid ethane to cool the grid more uniformly before a final plunge freezing step [60]. With these and future developments, cryo-EM is steadily pushing towards being a prevalent structural biology strategy. Solving structures to high resolution in a near-native state will greatly contribute to ISB.

### 2.5 Computational approaches

Despite the ever-increasing throughput of experimental methods for structure determination, structure prediction for new or engineered proteins from primary sequence remains an important and challenging problem. Computational structure prediction methods are most accurate when templates are available for proteins with a high sequence similarity [61]. These techniques, known as template-based modeling, match input sequences to evolutionarily-related homologs in the PDB [62], and use these homologs to predict likely folds in the input protein [63], [64]. As most unknown proteins likely have a similar homolog in the PDB for at least a portion of their sequence [65], this strategy is successful for a large portion of unknown proteins. Interactive websites that employ homology detection methods have been developed, allowing non-experts to generate 3D structure predictions for proteins of interest [63]. However, these approaches inherit several of the limitations of their training database. First, since protein structures are primarily determined through X-ray crystallography, computational predictions also represent snapshots of the protein in a crystalline state. Second, the accuracy of template-based models degrades when there is a lack of similar template proteins for the target structure [61]. Thus, structure predictions for proteins without a close resemblance to those with resolved 3D structure can be unreliable. While the first of these limitations might be resolved through the increased availability of protein structures in aqueous environments, data for these types of proteins is likely to remain limited in the near-term. As a result, methods for accurate template-free modeling will likely play an important role in the field of ISB.

Machine learning (ML) methods have rapidly advanced the prediction quality of template-free methods, as evidenced by the most recent critical assessment of protein structure prediction (CASP 13) [66]. In these challenges, predicted structures leveraging ML could achieve a high structural similarity (up to 60% global test distance) even for the most difficult proteins, as measured by the availability of similar templates (Fig. 2E). These methods take advantage of multiple sequence alignments (MSAs) as input features to predict residue-residue distance matrices. MSAs establish the evolutionary history of a given protein family and show where mutations of individual amino acids have occurred. Correlations between mutations are evidence of physical proximity in a folded protein, and thus clues to a protein family’s 3D structure are present in genomic libraries, even if no member of that protein family has had its structure resolved [67]. ML models for structure prediction typically take the form of deep neural networks with convolutional architectures [68]. Similar to techniques used in image processing, these models allow extraction and prediction of two-dimensional residue-to-residue features regardless of their location in the protein’s primary sequence. The best-performing method in CASP13, AlphaFold, used a combination of multiple neural network components and traditional physics-informed energy simulations from Rosetta in an interconnected prediction pipeline [69], [70]. However, limitations to these methods exist. MSA inputs require sufficient data for each protein family and are only able to give family-level predictions. Thus, engineered proteins, those with unique folds with respect to evolutionarily similar proteins, or those without highly conserved analogs in genomic databases are unlikely to be predicted accurately.

Future machine learning methods may address some of these limitations. Models based on neural network architectures from natural language processing have seen increased application in protein structure prediction. Similar to techniques in language translation, these models process sequences of input data to make sequences of predictions. These models implicitly use information from an MSA through unsupervised pretraining, with the benefit of being able to make individualized predictions for each member of a protein family. Recurrent neural networks have shown best-in-class performance for predicting remote homology, an important step in determining a protein’s 3D structure [71]. The transformer architecture, based on self-attention mechanisms, has quickly become a top-performing architecture in natural language processing [72]. Applications of transformer models to protein sequence data have shown that self-attention layers are able to learn useful properties from raw amino acid sequences [73]. Further development of these techniques for contact prediction is ongoing [74], and machine learning predictions for template-free structure prediction will likely get more accurate and specific.

Finally, the use of *sparse* data in protein structure prediction is still not widely considered in machine-learning based approaches [66]. Data-assisted modeling is likely to be especially important in interpreting the results of SAXS and single-particle EM, where experimental data alone is insufficient to fully constrain the protein structure. The typical strategy for incorporating sparse SAXS data is to generate a population of structure predictions using fast structure prediction models, and then use sparse data to down-select these predictions to those consistent with the experimental measurements [75]. In CASP13, the highest-performing deep learning methods outperformed those which use sparse data, likely due to low participation in the sparse data challenge [76]. However, machine learning algorithms are likely well-suited to take advantage of these data types in making predictions. The growing availability of NMR and SAXS data, or the development of data augmentation techniques to simulate sparse data given a fully resolved structure may improve the accuracy of these methods.

## 3 Protein complex identification and characterization *in vitro* via combinatorial approaches

Structural determination of protein complexes is often more difficult than that of individual proteins due to the inherent flexibility and low affinity interactions of the subunits. In the previous section, we described techniques to structurally characterize individual proteins and stable complexes. The next step of bottom-up ISB aims to address the problems of subunit flexibility and weak interactions by providing additional biophysical information about the interactions, which can be used to stabilize the complexes for further structural studies. This section will give a brief overview of three main types of protein interaction characterization: 1) identification of interacting partners, 2) modifications to capture complexes, and 3) combinatorial techniques to characterize interactions more accurately. The first two categories are the more traditional approach to protein complex characterization, while the third category is a more modern solution to the determination of transient interactions.

### 3.1 Identification of complexes and complex structure prediction

Arguably the most important step to characterizing complexes is identifying the components of interest which can be achieved using computational and experimental approaches. There exist many databases of known protein interfaces that can be used to identify protein complex partners [77], [78], [79], [80]. Recently, new computational techniques based on deep learning algorithms have led to the prediction of protein–protein interactions, enabling the identification of novel complexes, including some that might be transient, and that may have so far escaped researchers using more traditional techniques [81], [82], [83]. Experimentally, co-immunoprecipitation (co-IP) can be used to identify high-affinity complexes [84]. However, weak, transient interactions tend to be disrupted during the co-IP procedure due to thorough washing steps [4]. Instead, XL-MS or proximity labeling can be used to identify potential interactions of interest. XL-MS uses chemical crosslinkers that link specific surface exposed residues that come within a certain distance of each other, depending on the length of the cross-linker. The crosslinked proteins are proteolytically digested and analyzed by mass spectrometry to identify the proteins that form the complex [85]. Proximity labeling is more involved because it requires that the bait protein of interest be fused to BirA, which promiscuously biotinylates proteins that interact with the bait. The biotinylated prey can then be captured using a streptavidin pulldown and further analyzed by mass spectrometry [86]. When the components of the complex have been identified, binding surfaces and relative orientation of the partners can be predicted via molecular modeling. This is usually accomplished using protein–protein docking approaches such as ZDOCK, DOT, or RosettaDock among many others [87], [88], [89]. For some of these software programs, protein–protein fit can be further improved by allowing flexibility of the protein backbone [90]. The degree of success using these approaches can vary greatly depending on the complexity of the problem. Nevertheless, they can provide vital information to better understand the function of these complexes and generate key scientific hypotheses before experimental structures are available.

### 3.2 Isolation of stable protein complexes

Complexes can be chemically modified to stabilize interactions that would otherwise disrupt stable structures. If a protein interaction has a predicted interface, observed either from molecular docking or experimental confirmation, the complex could be locked into place by engineering a disulfide bridge. There are online servers such as Disulfide by Design [91], MODIP [92], SSBOND [93], and Yosshi [94] that predict which residues can be mutated to cysteines to create artificial bridges. However, most of these servers rely on prior structural knowledge, which is not always available. Without structural data, it is possible to use chemical crosslinkers to stabilize the complex of interest at interaction sites. Chemical crosslinking often has the drawback of creating large aggregates unless precautions are taken to limit the crosslinker interaction with the proteins such as AgarFix (where one immobilizes the protein of interest in agarose, washes with crosslinker, washes with buffer, and finally elutes the crosslinked sample from the agarose droplet) [95]. Some protein interactions can be stabilized enough for structural techniques by forcing proximity of the subunits. A straightforward way to increase hetero-oligomer subunit proximity is by physically tethering the components together with a flexible linker. These designed constructs can be expressed and purified similarly to the protocol used for isolating the individual proteins, without the need for additional binding tests [96]. However, physical fusion runs the risk of crosstalk between molecules if the binding affinity is higher than expected. This could result in aggregation of the protein sample, rendering it useless for structural studies. In the case of weak affinity homo-oligomers, creating a direct fusion protein is sometimes difficult to clone due to the repetition of the same gene. To address this, some groups have used indirect fusion partners, such as the small trimeric protein Foldon, to chaperone the formation of higher order oligomers [97]. For example, Foldon was fused to the spike protein of SARS-CoV-2 to maintain the trimeric structure when capturing one of the first cryo-EM structures released related to SARS-CoV-2 [98].

### 3.3 Additional characterization via ISB

Structural characterization of protein complexes is often of lower resolution than that of individual globular proteins, due to flexible subunits and incomplete complex formation. ISB is a means of supplementing traditional structural data with other sources to provide a more complete picture of the protein–protein interactions [7]. For example, in 2018, Kim *et al.* used ISB to fully characterize the *S. cerevisiae* nuclear pore. They combined XL-MS with fluorescent protein labeling to determine relative localization and stoichiometry of the nucleoporins (NUPs). Then, using prior structural data from crystal structures, SAXS, and integrative modeling, the structures of the NUPs were docked into a low-resolution cryo-electron sub-tomogram averaged map (described in the following section) of the nuclear pore, resulting in a detailed map of the 87 MDa complex [99]. The combination of techniques to create a composite structure has a unique site of the PDB, called the PDB-Dev where ISB structures can be deposited with a multitude of techniques listed as contributors to the structural information [100]. Frequently, these structures combine either SAXS or EM data, which contributes a general outline of the complex, with a computational model, which provides theoretical detailed structural information, confirmed by biochemical or biophysical means. For example, one could use protein mutagenesis to disrupt predicted protein–protein interactions, which would generate a smaller shell in SAXS or EM. For samples with flexible domains, one could use FRET, which labels two parts of the complex with different fluorophores –when the two parts are in proximity, the emission of one fluorophore donates energy to the second fluorophore resulting in an observable fluorescence emission signal [101]. Finally, recent advances in XL-MS have led to its use in the characterization of protein–protein interactions. These innovations in XL-MS include new variants of crosslinker available, forgoing limitations of the proximity of two lysine residues in the sample; 0-length crosslinkers, which do not have a spacer arm and can be used to locate close interaction partners, and improvements in computational analysis of XL-MS data [102].

## 4 Advancements in electron microscopy and computational biology contribute to the characterization of quinary interactions.

In the previous sections, we described methods used in bottom-up ISB, where protein interaction networks are built from individual structures and biophysical data. We now change our focus to look at more recent advances that can be used for a top-down approach to ISB. In top-down ISB, data from the whole cell are collected, followed by characterizing captured complexes with molecular details. Here, we describe the advantages and disadvantages to three cryo-EM techniques that could be used for top-down ISB.

### 4.1 Whole-cell cryo-electron tomography

**Fig. 3:**
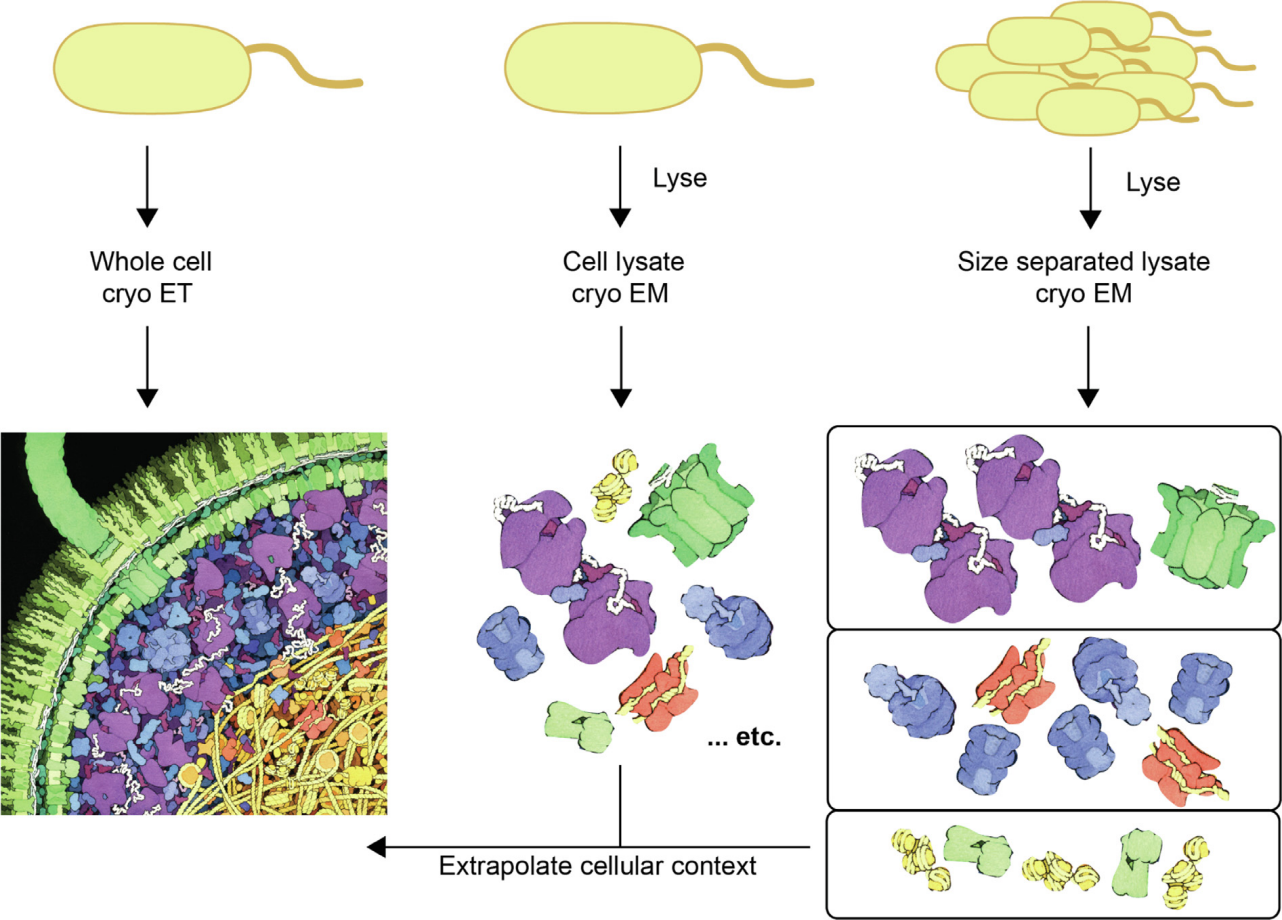
*in situ* structural characterization of quinary interactions. A) Cryo ET of an *E. coli* cell, resulting in a detailed look of the inside of the cellular interactions (illustration by David S. Goodsell, the Scripps Research Institute). B) Individual cell lysate applied directly to a cryo-EM grid results in a mixture of protein structures that have been minimally disrupted, as represented by the structures extracted directly from the cell picture. C) Separating the lysate of several cells using only size exclusion chromatography results in fractionated cryo-EM samples (represented by the particles boxed by physical size), which are computationally easier to process.

Logically, the most reliable way to characterize transient interactions in the cell would be to avoid disrupting the cell entirely. The best technique to examine these interactions at the nanometer, and potentially sub-nanometer scale, is whole-cell cryo-electron tomography (cryo-ET) (Fig. 3A). Performing Cryo-ET on a whole cell is similar to cryo-EM SPA, although the intact cell is captured in vitreous ice instead of an isolated protein [103]. Currently, whole-cell cryo-ET is limited to smaller cells, such as bacteria, or thin cellular regions, such as mammalian cell protrusions, due to the limitations to electron penetration and vitreous ice formation when a sample is thicker than 500 nm [104]. However, correlated light and electron microscopy (CLEM) can be performed in thicker cells, such as mammalian cells, to locate a region of interest to be isolated by cryo-focused ion beam (cryo-FIB) milling [103]. If there are many copies of the complex of interest, sub-tomogram averaging can be used to create a moderate resolution reconstruction of the complex [105]. Cryo-ET, in conjunction with sub-tomogram averaging has been used to determine the structures of the nuclear pore [106], ribosomes [107], viral capsids [108], S-layer proteins [109], and flagellar motors [110]. Yet, there are still limitations to this technique, which include: the need for cryo-FIB milling of thick samples which removes the surrounding cellular context, the need for many copies of a structure to enable near-atomic resolution reconstructions, and the loss of small proteins and complexes in the noise of the cellular milieu.

### 4.2 Single-cell cryo-EM SPA

To overcome some of the technical limitations of cryo-ET, the Braun group has developed the cryoWriter system in conjunction with the lysis of individual cells (Fig. 3B). The lysis of individual cells results in approximately 3 nl of lysate which can be applied directly to a grid to be viewed via the cryoWriter system, which uses 1000-fold less sample than current commercially available cryo-plunging devices [111]. This protocol has been used to look at the lysate of individual mammalian cells, which circumvents the need for cryo-FIB milling used in tomography. Using this system, combined with negative stain EM, they were able to identify many recognizable cellular components, including vault organelles, actin filaments, and proteasomes [112]. The technique was further expanded to examine the differences between control cells and heat-shocked cells, showing an upregulation of a protein with the predicted shape of a chaperone protein. The upregulation of the chaperone was characterized by the novel differential visual proteomics (DVP) algorithm which categorizes the differences in protein expression levels based on normalized particle counts from 2D classification in negative stain EM samples. Currently, DVP is limited by the software used to isolate and classify particles, which is more reliable for particles with distinct features and/or larger than 100 kDa. Furthermore, this technique requires a massive amount of data to account for low-abundance proteins correctly [113]. Therefore, DVP used in conjunction with individual cell lysis works well to remove some of the barriers presented by cryo-ET such as an ability to examine the contents of an entire mammalian cell, as well as a reduction in local noise to make identification of complexes simpler. Two disadvantages of this technique are that the lysis of the cell may serve to disrupt native interactions and the cryoWriter is not yet commercially available.

### 4.3 Cryo-EM SPA and XL-MS

Another promising technique that has been utilized to examine transient protein interactions is the combination of EM and XL-MS (Fig. 3C) wherein a batch of cells is lysed and then applied directly to a size exclusion column to separate cellular components based solely on size [114]. The fractionation of the sample decreases the noise in EM that is otherwise found in cryo-ET and single cell lysis. However, this technique is the most disruptive, combining the lysis of millions of cells, creating an average view of a population of cells, while potentially breaking transient interactions. To address the confounding factor of size exclusion purification of the lysate, the lysates are also subjected to XL-MS. Many papers have covered the experimental details of XL-MS – here we will only focus on its use in combination with EM [102], [115], [116], [117], [118]. In this instance, the crosslinker can be added either during growth, which will ensure that relevant quinary interactions can be captured but is limited to very specific crosslinkers that can cross the cell membrane. On the other hand, the crosslinker can be added immediately post-lysis, which gives a wider variety of potential crosslinkers, but with the disadvantage of capturing non-native interactions [102]. However, the data of interactions found from XL-MS can inform the structures determined by cryo-EM. As an example, Kastritis *et al*. used this combination of experiments to determine that fatty acid synthetase interacted with another protein in approximately 10% of the particles they observed, which their XL-MS data showed to be a carboxylase [114]. Therefore, this technique is useful for identifying transient interactions with less noise than some other techniques, but it is also the most disruptive to native interactions.

One disadvantage of all the above techniques is that quinary interactions are, by definition, less stable due to their transience. Thus, the structures determined may still be at a lower resolution due to the highly flexible nature of the complexes and lower frequency of observation during computational sorting. Further, cryo-EM of heterogeneous samples and whole-cell cryo-tomography are currently limited to lower resolution due to the complexity of the samples being studied. Strong modelling is necessary to interpret the data gained from all of the above structural techniques. Therefore, it is still necessary to employ structural techniques such as those described in the first and second section to determine high-resolution structures that can be docked into lower resolution maps generated by heterogeneous EM samples. Failing at determining the structure, structural models created computationally, such as those described in the first section, can be fit into low-resolution maps, as described in the second section.

## 5 Conclusion

**Fig. 4:**
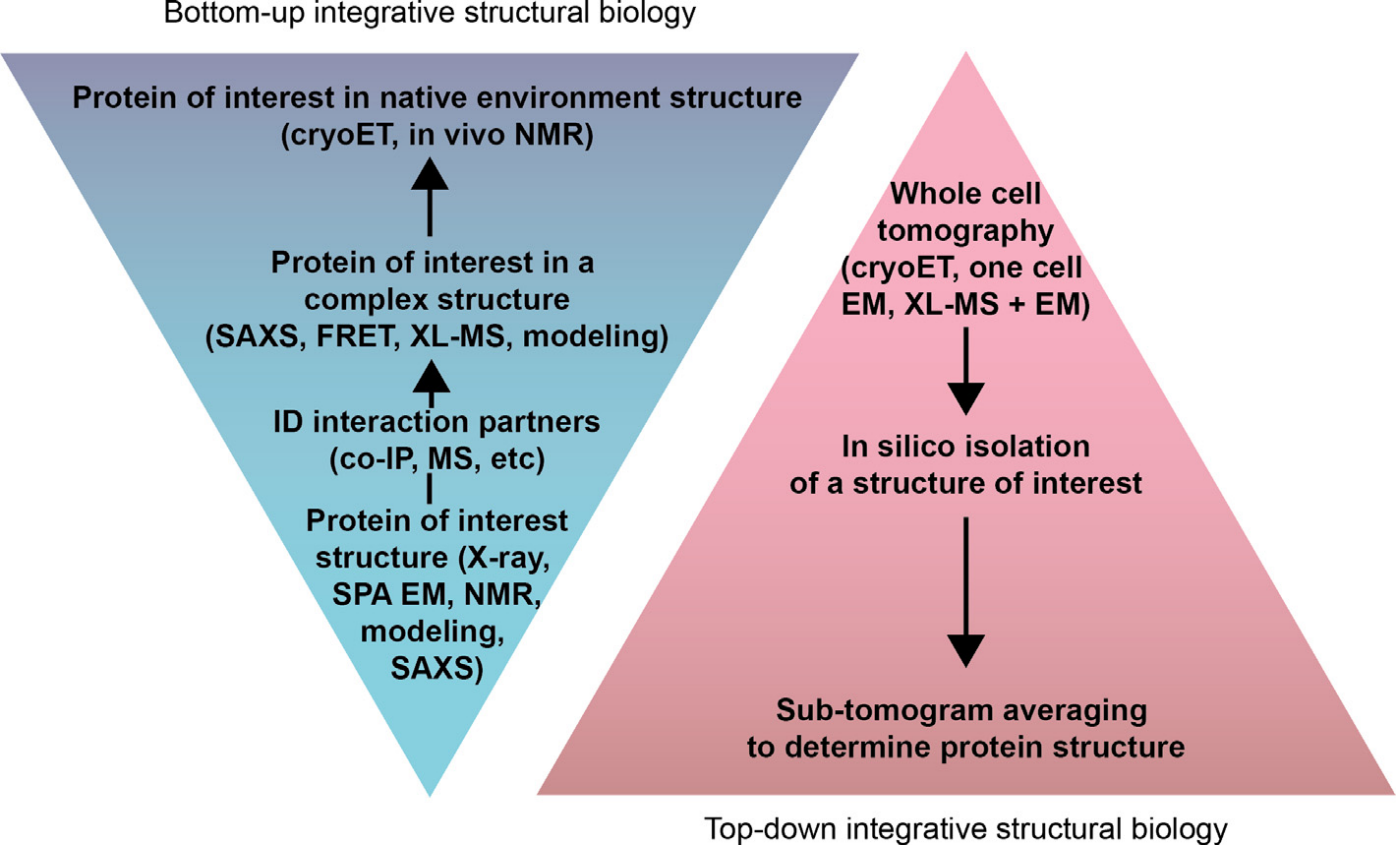
Integrative structural biology flow chart. Traditional ISB is performed from bottom-up (blue triangle), starting with structures of individual components and building up to stable complexes and quinary interactions. With recent developments, ISB is beginning to be done from top-down starting from whole cell tomography providing the densities to map individual complexes (red triangle). (For interpretation of the references to colour in this figure legend, the reader is referred to the web version of this article.)

The field of ISB is rapidly approaching the realization of whole-cell structural biology. This is in large part due to recent developments in structural biology, such as the cryo-EM resolution revolution and the increasing accuracy of computational protein structure prediction. When structures from individual proteins are combined with rigorous biochemistry and biophysical experiments, one can begin to build up protein complex structures in a bottom-up ISB approach, whether they are stable or transient (Fig. 4). These complexes can then potentially be contextualized with cryo-ET of the whole cell. It is also possible to start with cellular cryo-ET, combined with XL-MS and structural data to study protein complexes from the top-down (Fig. 4). Currently, determining cellular context reliably from noise in the sample is the limiting step to true cellular structural biology, although the rate of technological innovation in the electron microscopy field seems promising for ISB. Soon, we may be able to track transient protein interactions at atomic resolution through the entire life cycle of a cell. This advancement can help provide a better understanding of internal metabolism and stress responses in microorganisms and guide more systematic metabolic engineering campaigns.

## Declaration of Competing Interest

The authors declare that they have no known competing financial interests or personal relationships that could have appeared to influence the work reported in this paper.
